# Early liver biopsy, intraparenchymal cholestasis, and prognosis in patients with alcoholic steatohepatitis

**DOI:** 10.1186/1471-230X-11-115

**Published:** 2011-10-28

**Authors:** Laurent Spahr, Laura Rubbia-Brandt, Muriel Genevay, Antoine Hadengue, Emiliano Giostra

**Affiliations:** 1 Gastroenterology and Hepatology, Specialities Department, University Hospitals of Geneva, 4, Rue Gabrielle Perret-Gentil, CH-1211 Geneva, Switzerland; 2 Clinical Pathology, Pathology Department, University Hospitals of Geneva, 4, Rue Gabrielle Perret-Gentil, CH-1211 Geneva, Switzerland

## Abstract

**Background:**

Alcoholic steatohepatitis (ASH) is a serious complication of alcoholic liver disease. The diagnosis of ASH requires the association of steatosis, evidence of hepatocellular injury with ballooning degeneration, and polynuclear neutrophil infiltration on liver biopsy. Whether these lesions, in addition to other histological features observed in liver tissue specimens, have prognostic significance is unclear.

**Methods:**

We studied 163 patients (age 55 yrs [35-78], male/female 102/61) with recent, heavy (> 80 gr/day) alcohol intake, histologically-proven ASH (97% with underlying cirrhosis, Maddrey's score 39 [13-200], no sepsis), who had a liver biopsy performed 3 days [0-10] after hospital admission for clinical decompensation. A semi-quantitative evaluation of steatosis, hepatocellular damage, neutrophilic infiltration, periportal ductular reaction, intraparenchymal cholestasis, and iron deposits was performed by two pathologists. All patients with a Maddrey's score ≥ 32 received steroids. The outcome at 3 months was determined. Statistical analysis was performed using the Wilcoxon and Fisher's exact tests, Kaplan-Meier method, and the Cox proportional hazard model.

**Results:**

43 patients died after 31 days [5-85] following biopsy. The 3-month survival rate was 74%. Mean kappa value for histological assessment by the two pathologists was excellent (0.92). Univariate analysis identified age, the Maddrey's score, the Pugh's score, the MELD score and parenchymal cholestasis, but not other histological features, as factors associated with 3-month mortality. At multivariate analysis, age (p = 0.029, OR 2.83 [1.11-7.2], intraparenchymal cholestasis (p = 0.001, OR 3.9 [1.96-7.8], and the Maddrey's score (p = 0.027, OR 3.93 [1.17-13.23] were independent predictors of outcome. Intraparenchymal cholestasis was more frequent in non survivors compared to survivors (70% versus 25%, p < 0.001). Serum bilirubin was higher in patients with severe compared to those with no or mild intraparenchymal cholestasis (238 [27-636] versus 69 [22-640] umol/l, p < 0.001).

**Conclusions:**

In this large cohort of patients with histologically documented ASH early after admission and no sepsis, liver biopsy identified marked intraparenchymal cholestasis as an independent predictor of poor short term outcome together with age and the Maddrey's score. It may be hypothesized that incorporation of this particular variable into existing disease severity scores for ASH would improve their performance.

## Background

Alcoholic steatohepatitis (ASH) is an acute inflammatory liver disease associated with a poor outcome [1]. In patients with a severe form of ASH, as defined by a Maddrey's score (also reported as Maddrey's discriminant function) ≥ 32, a 4-week course of corticosteroids significantly reduces the short-term mortality by approximately 25% [2].

Decompensated cirrhosis is a common clinical presentation of ASH in Western Europe [1], but the diagnosis of ASH using only clinical and biological criteria is very challenging for the physician. Accordingly, ASH but also other situations such as infection, gastrointestinal bleeding, or drug-induced hepatitis are common precipitants of acute deterioration in patients with alcoholic cirrhosis, a condition referred to as acute-on-chronic liver failure [3]. Therefore, when a severe form of ASH is suspected, as assessed by the Maddrey's discriminant function [2] or the MELD score [4], a liver biopsy is strongly recommended to make a definite diagnosis and to guide steroid therapy.

Histologically, ASH is defined by the presence of steatosis (macro- and/or microvesicular), hepatocellular injury (ballooning, apoptosis), and infiltration of the liver lobule by polynuclear neutrophils[5]. Mallory-Denk hyaline bodies, megamitochondria, perisinusoidal fibrosis, mild iron deposits, some degree of ductular reaction (also described as cholangiolar proliferation corresponding to proliferation of hepatic progenitors[6]), and intraparenchymal cholestasis are also described[7,8] but not required for diagnosis. Equivalent terms used for intraparenchymal cholestasis include bile pigments accumulation, bilirubinostasis, intralobular cholestasis or cholestatic alcoholic hepatitis[6,7,9]. Liver biopsy is mostly performed in patients with presumed ASH to exclude other causes of liver disease and to confirm the presence of ASH. Whether the full spectrum of histological alterations observed in liver biopsies of patients with ASH has a clinical significance remains unclear[7]. Thus, the aim of this study was to explore the prognostic value of several histological features observed on liver biopsy performed early after hospital admission in a large number of patients with recent active alcohol intoxication, documented ASH and no associated sepsis.

## Methods

### Patients

**Figure 1:**
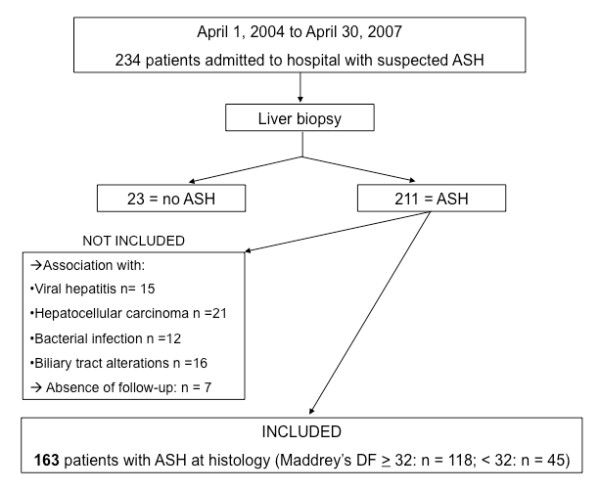
**Flowchart of patients' selection**. Abbreviation: DF: discriminant function.

The study population included 163 patients with ASH admitted to our institution between April 2004 and April 2007, selected according to the algorithm provided in Figure 1. One hundred and forty two patients were selected from control arms of previous interventional or observational cohorts[10-12] and 21 patients had a liver biopsy performed as part of the diagnostic work-up of an acute deterioration of liver function in patients with alcoholic liver disease. In all cases, the purpose of liver biopsy was to confirm a presumed diagnosis of ASH.

To be eligible, patients had to present with a history of heavy recent alcohol intake (> 80 gr per day), no radiological evidence of bile duct alterations, no documented hepatocellular carcinoma, no positive serology for hepatitis A, B, C or HIV, no disease associated with iron overload and no documented infection at the time of hospital admission and of liver biopsy. The main reason for excluding patients with concomitant infection is the commonly accepted role of sepsis-associated cholestasis[13] as a confounding factor for the histological analysis. In addition, infection in decompensated cirrhotic patients with ASH is a major determinant of poor outcome [14]. Therefore, infection was actively sought in all patients at time of hospital admission, as recommended [15]. The evaluation included a diagnostic paracentesis, urine and blood cultures, and a chest radiograph. A neutrophil count > 250/mm^3 ^in ascites, whether or not associated with a positive culture, was diagnosed as a spontaneous bacterial peritonitis and considered an exclusion criterion. Similarly, patients with microbiological evidence of infection in urine or blood, or with a pulmonary infiltrate on chest X-ray were not eligible for the analysis. During follow-up, patients were monitored closely for signs of infection, in particular for spontaneous bacterial peritonitis as the most common site of infection in this patient population [15] and received immediate broad-spectrum antibiotics in case of confirmation of diagnosis. Patients who received steroids were also administered preventive antibiotherapy with daily oral norfloxacin, in accordance to local guidelines. The diagnosis of ASH was based on histology in all patients, and the biopsy was performed early after hospital admission. All patients received standard medical care including nutritional therapy and support regarding alcohol abstinence during follow-up, as recommended[16]. Patients with ASH and a Maddrey's score ≥ 32 or a MELD score ≥ 18 were identified as high risk for short-term mortality[4,17] and were all treated by prednisone 40 mg daily for 28 days[2] in addition to standard medical therapy. Patients' characteristics are summarized in table 1.

**Table 1:** Patients characteristics

variable	n	median	range
Gender (M/F)	102/61		
Age (yrs)		55	35-78
Child-Pugh's score		10	6-14
MELD score		18.5	7-37
Maddrey's score		39	13-200
Serum bilirubin (μmol/l)		91	17-636
Blood leucocytes (G/l)		7.4	5-18.3
Neutrophils (G/l)		6.2	3.8-15.2
ASAT (IU/l)		90	47-192
Alkakine phosphatase (IU/l)		160	89-239

During this period, 71 patients with ASH were not included due to concomitant bacterial infection identified at hospital admission (n = 12), coexistent viral hepatitis or HIV (n = 15), radiological evidence of bile duct disease (n = 16), multifocal hepatocellular carcinoma (n = 21) or because of incomplete follow-up (n = 7)(see Figure 1).

### Liver biopsy

It is our policy to perform a liver biopsy early in the course of hospitalization in patients with decompensated alcoholic liver disease and suspicion of ASH.

The liver biopsy was performed after a median time of 3 days after admission (range: 0-10 days), either percutaneously (Hepafix Liver biopsy set 17G, Braun Melsungen, Germany) or via the transjugular route (TJL-101-ET needle set, Cook Europe, Bjaeverskov, Denmark) depending on the presence of coagulopathy and/or ascites. The liver biopsy specimen was placed into formalin 10%, then fixed and embedded in a paraffin wax block to be processed for light microscopy. Serial sections were stained with haematoxylin-eosin, reticulin, Masson Trichrome and Pearl's coloration for iron. The entire liver biopsy specimen was examined by using 20-50 high-power fields (x 400), as previously described[18]. Histopathological studies were performed by two pathologists (LRB and MG) experts in liver diseases, who were unaware of patients' characteristics and outcome. The diagnosis of ASH was based on the coexistence of steatosis, ballooning degeneration of hepatocytes, and lobular infiltration with polynuclear neutrophils[5]. Additional features including fibrosis, Mallory-Denk hyaline bodies, iron deposits, ductular proliferation in the periportal areas, and intraparenchymal cholestasis were also carefully examined. We restricted our analysis of cholestasis to the lobular area, however we did not consider ductular bilirubinostasis in the periportal areas as it has been previously described to be associated with sepsis[13]. Thus, our analysis was focused on the presence of bile pigments (or bile plugs) in hepatocytes.

We assessed the intensity of individual lesions using a semi-quantitative score. Thus, a score of 1 or 0 was given if the histological lesion was present in > 50% or < 50% of all microscopic fields, respectively. Due to the possible influence of subjective interpretation by observers, the two histopathologists were asked to determine lesion intensity for each biopsy specimen in a blinded manner.

We arbitrarily chose to group hepatocyte ballooning and Mallory-Denk hyaline bodies under the term "hepatocellular damage" and macrovesicular and microvesicular steatosis under the term "steatosis". Due to the high prevalence (> 95%) of cirrhosis in our patient population, we decided not to take fibrosis into consideration. Thus, the following histological features were considered for the analysis: steatosis, hepatocellular damage, neutrophilic infiltration, ductular reaction, intraparenchymal cholestasis, and iron deposits.

### Biological values

All scores (Child-Pugh's score, Maddrey's and the MELD score) were obtained using variables measured at the time of liver biopsy. The variables taken into consideration in these scores include coagulation parameters and serum bilirubin, as well as creatinine in the MELD score[1]. Serological status for hepatitis A, B and C was determined at admission. Patients with excessive iron deposits on liver biopsy were studied for the C282Y HFE gene mutation to rule out hereditary hemochromatosis.

We used TNFα and soluble form of the receptor 1 for TNFα (sTNF-R1) serum values measured in a subgroup of patients from a previous study[11] to explore the relationship between these pro inflammatory cytokines and intraparenchymal cholestasis.

### Outcome

We determined the patients' clinical outcome over a 3-month follow-up period starting from the time of liver biopsy, using hospital records and clinical information obtained from the general practitioners. In case of death, the cause was identified.

### Ethical considerations

The study protocol was approved by the Institutional Review Board of the Hôpitaux Universitaires de Genève who allowed us to retrospectively analyze the histological, biological and clinical data of the study population.

## Statistical analysis

Variables are given either as categorical (histological features) or continuous (age, bilirubin) values, and expressed as median and ranges. Rating for continuous variables was determined according to median value in the group. The Wilcoxon and Fisher's exact tests were used to compare variables between groups. Inter-rater agreement for qualitative variables was assessed with K statistics. A kappa value greater than or equal to 0.75 was considered to represent good agreement. Patients' survival was analyzed by the Kaplan-Meier method. The prognostic significance of the variables in the univariate analysis was determined using the log rank test. Rating for each variable was determined according to the median value in the group. To identify independent predictors of mortality at 3 months, we performed a multivariate logistic regression analysis using a Cox proportional hazard model. A 2-sided P value of < 0.05 was considered statistically significant. All statistical tests were performed by using the Statistical Package for the Social Sciences, version 10.0 for Windows (SPSS, Chicago, IL, USA).

## Results

### Outcome

Thirty patients (18%) returned to some degree of alcohol consumption during follow-up. Overall survival rate in this cohort of patients with predominant severe ASH was 74%. Over a 3-month period following liver biopsy, 43 patients, including 39 with severe ASH, died at a median time of 31 days (range: 4-85 days) following liver biopsy. The causes of death were progressive liver insufficiency (n = 19), infection (n = 11), portal hypertensive bleeding (n = 8), and others (n = 5).

### Histological features

**Figure 2:**
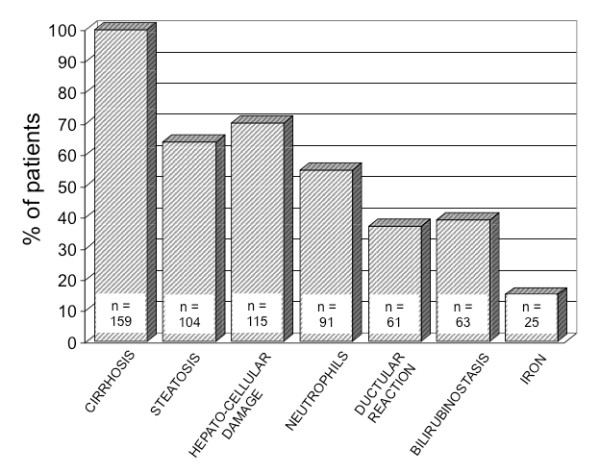
**Prevalence (expressed in percent of patients) of histological features that were severe in intensity, observed in 163 patients with ASH**.

**Figure 3:**
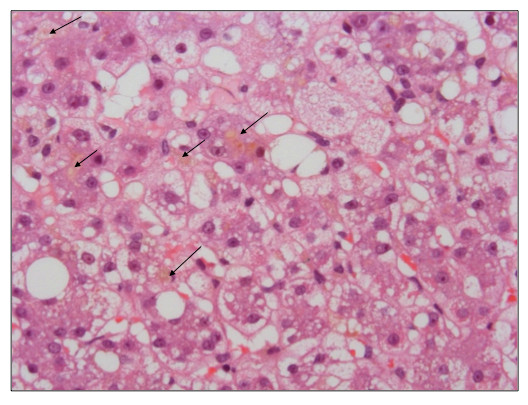
**Representative illustration of marked intraparenchymal cholestasis in a patient with alcoholic steatohepatitis (Haematoxylin-eosin stain, original magnification × 400)**. Arrows indicate bile plugs located in the liver parenchyma.

The liver biopsy was performed percutaneously in 31 patients and *via *the transjugular route in 132 patients, without major complications. Despite a high rate of tissue fragmentation due to cirrhosis in the vast majority of cases, the mean size of liver biopsy specimens was 19 [13-38] mm in length, allowing an accurate histological interpretation in all cases[19]. All patients fulfilled the criteria for ASH[5]. Additional features observed in > 50% of the biopsy specimen, and therefore considered to be severe according to our definition, are summarized in Figure 2. Thus, 97% of patients had severe fibrosis reaching the stage of cirrhosis, 64% demonstrated major steatosis, 70% had extensive hepatocellular damage, 55% showed important neutrophilic infiltrates, 37% had a marked periportal ductular reaction, 39% presented with severe intraparenchymal cholestasis, and 15% showed increased iron deposits that predominated in non parenchymal cells. Typical appearance of severe intraparenchymal cholestasis is illustrated in Figure 3.

**Figure 4:**
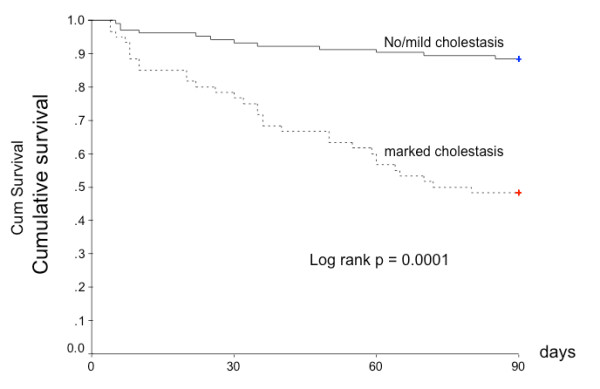
**Kaplan-Meier survival curve according to severity of intraparenchymal cholestasis on liver biopsy from 163 patients with ASH**.

Histological characteristics of survivors and non survivors are provided in table 2. Intraparenchymal cholestasis, but no other histological features, was significantly more frequent in patients with ASH who died during follow-up as compared to survivors. Cumulative survival according to severity of intraparenchymal cholestasis is illustrated in Figure 4.

**Table 2:** Histological features on liver biopsy according to clinical outcome

	Survivors (n = 120)	Non survivors (n = 43)	p value
Steatosis	69 (58%)	35 (81%)	0.4
Polynuclear neutrophils	66 (66%)	25 (60%)	0.9
Hepatocellular damage	80 (69%)	35 (81%)	0.8
Ductular reaction	47 (40%)	14 (32%)	0.9
Intraparenchymal cholestasis	31 (25%)	32 (70%)	0.001
Iron deposits	16 (14%)	9 (21%)	0.5

### Univariate analysis

Univariate analysis (table 3) identified age, the Maddrey's score, the Pugh's score, the MELD score and parenchymal cholestasis, but not ASAT, alkaline phosphatase, nor other histological features, as factors associated with mortality at 3 months.

**Table 3:** Univariate analysis

Variable	Cut-off	P value
Age (yrs)	≥ 50	
	< 50	0.01
Pugh's score	≥ 10	
	< 10	0.002
MELD score	≥ 19	
	< 19	0.001
Maddrey's score	≥ 32	
	< 32	0.001
Parenchymal cholestasis	≥ 50%	
	< 50%	0.001
Steatosis	≥ 50%	
	< 50%	0.213
Neutrophilic infiltration	≥ 50%	
	< 50%	0.320
Hepatocellular damage	≥ 50%	
	< 50%	0.054
Ductular reaction	≥ 50%	
	< 50%	0.628
Iron deposits	≥ 50%	
	< 50%	0.137
ASAT (IU/L)	≥ 90	
	< 90	0.26
Alkaline phosphatase (IU/L)	≥ 160	
	< 160	0.83

Abbreviations: ASAT: aspartate amino transferase

### Multiple regression analysis

Continuous (age, serum creatinine, bilirubin) and categorical variables (histological features), were incorporated into the statistical model. As the Maddrey's score determined steroid treatment in all patients, treatment with steroids *per se *was not included in the model as a variable. Results are given in table 4. At multivariate analysis, age (p = 0.029, OR 2.83 [1.11-7.2] and histological cholestasis (p = 0.001, OR 3.9 [1.95-7.8] were independent predictors of outcome.

**Table 4:** Multivariate analysis

Variable	P	OR	95% CI
Age	0.029	2.83	1.11-7.2
Intraparenchymal cholestasis	0.001	3.9	1.96-7.8
Maddrey's score	0.027	3.93	1.17-13.23

### Correlations

**Figure 5:**
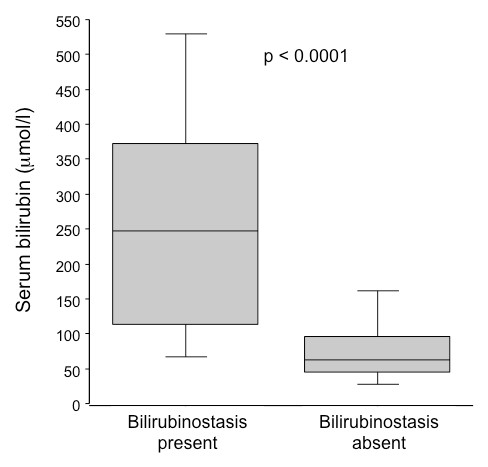
**Serum bilirubin levels according to the presence or absence of marked bilirubinostasis on liver biopsy**.

Patients with ASH and marked histological cholestasis on liver biopsy demonstrated higher serum bilirubin values compared to those without histological cholestasis (238 [27-636] vs 69 [22-640] μmol/l, p < 0.0001). Although the difference was highly significant, serum bilirubin values were distributed over a wide range in patients with marked lobular cholestasis (see Figure 5), and 27 patients (16.5%) with serum bilirubin exceeding 100 umol/l had no or mild cholestasis.

Serum values of TNFα and sTNF-R1 were available in 20 patients with cholestasis and 31 patients without cholestasis. In these patients, neither TNFα (7.1 [2-13] vs 5.3 [1-17.3] pg/ml, p = 0.53) nor sTNF-R1 (4600 [1000-8400] vs 2400 [300-7000] pg/ml, p = 0.15) serum values were different between groups.

## Discussion

The prominent finding of this large retrospective study is the presence of marked intraparenchymal cholestasis on liver biopsy as an independent predictor of survival, along with age and the Maddrey's score. Among other histological lesions commonly observed in ASH[20] bilirubinostasis was also the sole predictor of outcome. Interpretation of intrahepatic cholestasis is especially challenging in patients with decompensated cirrhosis at risk of developing biliary tract disease, infection or sepsis. In the latter situation, intrahepatic cholestasis is a prominent finding[13]. Having reasonably excluded the role of bile duct lesions or concomitant sepsis with the complete work-up performed at admission, intraparenchymal cholestasis can be considered as a lesion associated with ASH, as previously suggested[21]. Our results are in line with the study by Nissenbaum et al.[9] who reported lobular cholestasis in 38% of patients that correlated to malnutrition and a poor clinical outcome. In a recent cohort of alcoholics presenting as acute-on-chronic liver failure[6], a majority of whom reached the histological criteria for ASH, bilirubinostasis within the ductular reaction predicted in-hospital mortality. In this study, however, infection was documented in a substantial number of patients at time or very early (< 48 h) after hospital admission, and thus a role for sepsis in the development of intrahepatic cholestasis is difficult to rule out[13]. In our study, having excluded patients with sepsis at admission and focused our analysis to cholestasis in the liver lobule, we believe our data truly reflect lesions associated with ASH. Nevertheless, eleven patients (4%) developed infection during follow-up and died of sepsis, a frequent complication of severe ASH treated with steroids[15]. This incidence is low compared to recent data[14], and may result from a proportion of non severe ASH in our cohort as well as the implementation of antibioprophylaxis in steroid-treated patients.

The development of jaundice and elevated serum bilirubin is common in decompensated alcoholic liver disease associated with ASH[1]. Accordingly, serum bilirubin is integrated in several prognostic models[4,17,22] that guide therapeutic decisions. In our study, most patients with high serum bilirubin, but not all, showed marked lobular cholestasis. Nevertheless, our data are in line with results from previous studies[6,9,21] suggesting that histology provides additional prognostic information.

The pathophysiology of cholestasis in ASH is still uncertain. Proposed mechanisms include compression of intrahepatic biliary radicals by swollen and ballooned hepatocytes[23] or interference with basolateral and intracellular transport of bile acids[24] which may in turn induce cholestasis[25]. Accordingly, a particular bile acid pattern (elevated chenodeoxycholic and low deoxycholic acid serum levels) has been reported in patients with ASH [26], that correlated with histological features in general, but not with cholestasis in particular. This apparent dissociation between serum bile acids, bilirubin and tissue cholestasis has been observed in almost forty percent of the cohort described by Nissenbaum et al.[9]. Supplementation of ursodeoxycholic acid in patients with alcoholic cirrhosis and high serum bilirubin is associated with a decrease in biological tests of cholestasis [27] but histological changes are not described. Whether intracellular and canalicular trafficking of bile may be altered in ASH is a valid hypothesis. Both sepsis and ASH are characterized by a dysregulated inflammatory reaction with production of pro-inflammatory cytokines[10,16,28]. In both conditions endotoxemia may be detectable, TNFα is elevated, and bile flow is impaired at the cellular level[13,29,30]. In a subgroup of our patients, however, we were unable to demonstrate any relationship between bilirubinostasis and TNFα serum levels. Whether serum levels of cytokines truly reflect their biological activity within the hepatocyte is questionable.

Except for lobular cholestasis, other histological features were not associated with clinical outcome. Steatosis results from recent, heavy alcohol intake[16,20]. Major steatotic changes were present in the majority of patients, associated with recent and heavy alcohol intoxication. In alcoholic liver disease, the severity of steatosis predicts the development of cirrhosis over a 4-year period[31], but the short term significance of this feature in ASH is unknown. In our personal experience, steatosis is reduced and even disappears within weeks following decompensation in case of sustained alcohol abstinence. Marked hepatocellular damage as assessed by frequent Mallory-Denk bodies and ballooned hepatocytes was highly prevalent (> 70%) but not significantly related to short term mortality (p = 0.086). In a recent study on 54 alcoholic patients presenting as acute-on-chronic liver failure [32], the majority of whom were admitted to the intensive care unit and developed infection, the presence of Mallory-Denk bodies and ductular bilirubinostasis were predictors of in-hospital mortality. In a large cohort of patients with ASH treated with steroids, Mathurin et al [17] reported a better 1-year survival in patients with marked liver neutrophilic infiltration compared to those without this histological finding. Our results didn't confirm this association, and we hypothesize that an older age (55 years in our study versus 48 years in the study by Mathurin[17]), previously suggested to play a role in glucocorticoid efficacy, as well as inclusion of 27% of patients with non severe ASH in our cohort may explain this difference. Elevated liver iron is associated with accelerated fibrosis in alcoholics [33] and increased long term mortality in patients with alcoholic cirrhosis [34]. However, over a short time period in patients with ASH and cirrhosis, marked iron deposits didn't influence survival. Hepatocellular regeneration is an important point to consider in the evolution of decompensated liver disease. Extensive necrosis and apoptosis, replicative senescence of mature hepatocytes, toxicity of alcohol and metabolites, deleterious effect of cholestasis[35], may all participate in poor organ repair mechanisms in cirrhosis with superimposed ASH[36]. The intensity of the ductular reaction in the periportal region, considered as representative of regeneration processes[37], was not able to discriminate survivors and non survivors, consistent with recent data[6].

The strengths of our study is a detailed analysis of a large group of patients with a well-defined alcoholic liver disease based on histological findings obtained very early after hospital admission, presenting either in a severe or non severe form of ASH, in whom we described the outcome 3 months after clinical decompensation. Having performed a semi-quantitative analysis of the severity of lesions, we demonstrated a significant correlation between one of these features and clinical outcome. Accordingly, intrahepatic cholestasis is an independent predictor of 3-month mortality, together with age and the Maddrey's score, a commonly used robust parameter that includes serum bilirubin and prothrombin time[38]. We acknowledge that our study suffers from some limitations. First, due to missing date, we were not able to include modern prognostic markers such as the Lille[22] or the Glasgow[39] scores, nor the C-reactive protein. Whether intraparenchymal cholestasis combined with the short-term evolution of serum bilirubin included in the Lille score brings additional information on prognosis remained to be explored. Secondly, our assessment of intrahepatic cholestasis may be suboptimal with regards to the semi-quantitative analysis, and the criticism may be raised that conversion of a subjective observation (i.e. lesion present in more or less than 50% of the biopsy) into a categorical variable is arbitrary and subject to inconsistency. However, we have tested the reproducibility of the scoring system and demonstrated an excellent inter observer agreement between the two histopathologists. Thirdly, the prevalence of histological lesions in alcoholic liver disease may be influenced by the timing of liver biopsy in relation to clinical deterioration, as features such as steatosis, hepatocyte ballooning and Malloy-Denk bodies tend to be less prominent over time[40]. Therefore, it is questionable whether intralobular cholestasis would still be relevant if the liver biopsy had been performed later in the course of the disease.

## Conclusion

This detailed analysis of histological features observed on liver biopsies of patients with ASH demonstrates that among the full spectrum of lesions observed in alcoholic liver disease, only marked intralobular cholestasis is closely associated with the short term clinical outcome. It may be hypothesized that incorporation of this variable into existing disease severity scores for ASH would improve their performance.

## List of Abbreviations

ASH: alcoholic steatohepatitis; MELD: model for end-stage liver disease; Maddrey's DF: Maddrey's discriminant function; INR: international normalized ratio; TNFα: tumor necrosis factor alpha

## Competing interests

The authors declare that they have no competing interests.

## Authors' contributions

LS designed and conceived the study, performed liver biopsies, was responsible for acquisition and interpretation of data, drafted the manuscript. LRB participated to the conception of the study, interpreted liver biopsy, drafted the manuscript. MG participated to the conception of the study, performed liver biopsy interpretation, helped to draft the manuscript. AH participated to the conception and search for funding of the study, helped to draft the manuscript. EG participated to the conception and coordination of the study, performed the statistical analysis, participated to draft the manuscript.

All authors read and approved the final version of the manuscript.

## Acknowledgements and funding

This study was supported by an unrestricted grant from the Foundation for Liver and Gut Studies (FLAGS) in Geneva. The authors thank Nicolas Goossens for his help in proof reading the manuscript.

## Pre-publication history

The pre-publication history for this paper can be accessed here:

http://www.biomedcentral.com/1471-230X/11/115/prepub
